# Risk factors analysis and survival prediction model establishment of patients with lung adenocarcinoma based on different pyroptosis-related gene subtypes

**DOI:** 10.1186/s40001-023-01581-x

**Published:** 2023-12-18

**Authors:** Ziang Wen, Bei Pei, Longfei Dai, Peng Lu, Xiangyu Li, Chengxin Zhang, Shenglin Ge

**Affiliations:** 1 Department of Cardiovascular Surgery, The First Affiliated Hospital of Nanjing Medical University, Nanjing, China; 2 The Graduated School, Anhui University of Traditonal Chinese Medicine, Hefei, China; 3 The Graduated School, Anhui Medical University, Hefei, China; 4 Department of Cardiovascular Surgery, The First Affiliated Hospital of Anhui Medical University, Hefei, China

**Keywords:** Pyroptosis, Lung adenocarcinoma, Gene, Signature, Survival

## Abstract

**Background:**

Lung adenocarcinoma (LUAD) is a common cancer with a poor prognosis. Pyroptosis is an important process in the development and progression of LUAD. We analyzed the risk factors affecting the prognosis of patients and constructed a nomogram to predict the overall survival of patients based on different pyroptosis-related genes (PRGs) subtypes.

**Methods:**

The genomic data of LUAD were downloaded from the TCGA and GEO databases, and all data were filtered and divided into TCGA and GEO cohorts. The process of data analysis and visualization was performed via R software. The data were classified based on different PRGs subtypes using the K-means clustering method. Then, the differentially expressed genes were identified between two different subtypes, and risk factors analysis, survival analysis, functional enrichment analysis, and immune cells infiltration landscape analysis were conducted. The COX regression analysis was used to construct the prediction model.

**Results:**

Based on the PRGs of LUAD, the patients were divided into two subtypes. We found the survival probability of patients in subtype 1 is higher than that in subtype 2. The results of the logistics analysis showed that gene risk score was closely associated with the prognosis of LUAD patients. The results of GO analysis and KEGG analysis revealed important biological processes and signaling pathways involved in the differentially expressed proteins between the two subtypes. Then we constructed a prediction model of patients’ prognosis based on 13 genes, including IL-1A, P2RX1, GSTM2, ESYT3, ZNF682, KCNF1, STK32A, HHIPL2, GDF10, NDC80, GSTA1, BCL2L10, and CCR2. This model was strongly related to the overall survival (OS) and also reflects the immune status in patients with LUAD.

**Conclusion:**

In our study, we examined LUAD heterogeneity with reference to pyroptosis and found different prognoses between the two subtypes. And a novel prediction model was constructed to predict the OS of LUAD patients based on different PRGs signatures. The model has shown excellent predictive efficiency through validation.

**Supplementary Information:**

The online version contains supplementary material available at 10.1186/s40001-023-01581-x.

## Introduction

Lung cancer (LC), the most common cancer worldwide, is a significant cause of cancer death [1]. Non-small cell lung cancer (NSCLC) accounts for the majority of LC, and lung adenocarcinoma (LUAD) accounts for more than half of NSCLC [2]. Despite the excellent surgical outcome and prognosis of early-stage LUAD, the overall prognosis of LUAD is still poor. Comprehensive therapy, represented by chemotherapy such as platinum drugs and immune checkpoint blockade therapy such as PD-1 and PD-L1 inhibitors, has shown promising benefits in advanced LUAD [3, 4]. Unfortunately, due to the widespread tumor heterogeneity, a large number of patients are resistant to the medication, leading to cancer death [5, 6]. Therefore, it is necessary to discover novel subtypes of LUAD to predict the overall survival and provide more appropriate therapy options for the patients.

Pyroptosis is a type of programmed cell death that can promote inflammation. It is characterized by two major features, cell swelling and rupture, and the release of a range of inflammatory factors [7, 8]. Pyroptosis is strongly associated with various cancer, such as gastric cancer, esophageal cancer, and lung cancer [9–13].

Activation of pyroptosis has been found to have an impressive inhibitory effect on lung cancer. Cucurbitacin B can inhibit NSCLC through activation of TLR4/NLRP3/GSDMD-dependent pyroptosis [14]. Polyphyllin VI exerts anti-tumor effects by regulating the ROS/NF-κB/NLRP3/GSDMD signaling axis [15].

In our study, we constructed a model based on different PRGs signatures to predict the overall survival rate using the gene expression datasets from the TCGA and GEO databases. Our findings can guide individualized treatment and prognosis prediction of LUAD patients.

## Methods

### Data collection

The TCGA data of LUAD samples were collected from Genomic Data Commons. The Gene Expression Omnibus (GEO) data of LUAD sample were also downloaded (GSE31210). Both TCGA and GEO databases were used to obtain normalized gene expression data and clinical information for further analysis. R software was used for validation and visualization of the data.

### Construction of subtypes

According to the previous studies, we selected 52 pyroptosis-related genes (PRGs) [16, 17]. We compared the differential expression of PRGs in both normal and tumor tissues. K-means clustering method was used to classify the samples into distinct molecular subtypes based on different PRGs signatures via the “ConsensuClusterPlus” R package. The “Survival” R package was also used to perform a prognostic analysis on different subtypes of samples. The differentially expressed genes (DEGs) of different subtypes were found using the “limma” R package. A heatmap was constructed for comparison of differential expression of PRGs in normal and LUAD tissues.

### Establishment and validation of prognostic signature

Based on P value and fold changes (FC), we used R software to identify DEGs associated with PRGs from the TCGA cohort. The univariate Cox method was used to identify DEGs related to the prognosis from both TCGA and GEO cohorts. The least absolute shrinkage and selection operator (LASSO) logistic regression and the “glmnet” package were used to perform feature selection of screening diagnostic biomarkers for different subtypes LUAD. The “survival” and “survminer” R packages were used to compare the OS between high-risk and low-risk groups in two cohorts. The ROC curves were plotted to access the predictive value of prognosis using the ‘‘timeROC’’ R package. The PCA and t-SNE analyses were performed on all data in the TCGA and GEO cohorts using the “Rtsne” and “ggplot2” R packages.

Then, we used univariate and multivariate Cox regression analyses to assess the prognosis significance and identified the risk factors associated with prognosis based on the PRGs signature. Similarly, a nomogram was constructed to visualize the survival probability of patients based on the risk factors. A calibration curve was drawn to assess the accuracy of the nomogram we built.

### Functional enrichment analysis

The “ClusterProfiler” and “EnrichPlot” R packages were used to perform GO and KEGG analyses on DEGs of two subtypes in TCGA cohort. The “GSVA” R package was used to identify the immune cell scores and the activities of immune-related pathways of patients in both TCGA and GEO cohorts.

### Statistical analysis

R software (version 4.1.1) was used for all statistical analyses. P value < 0.05 was considered statistically significant.

## Results

### Identification of different pyroptosis-related subtypes

**Fig. 1:**
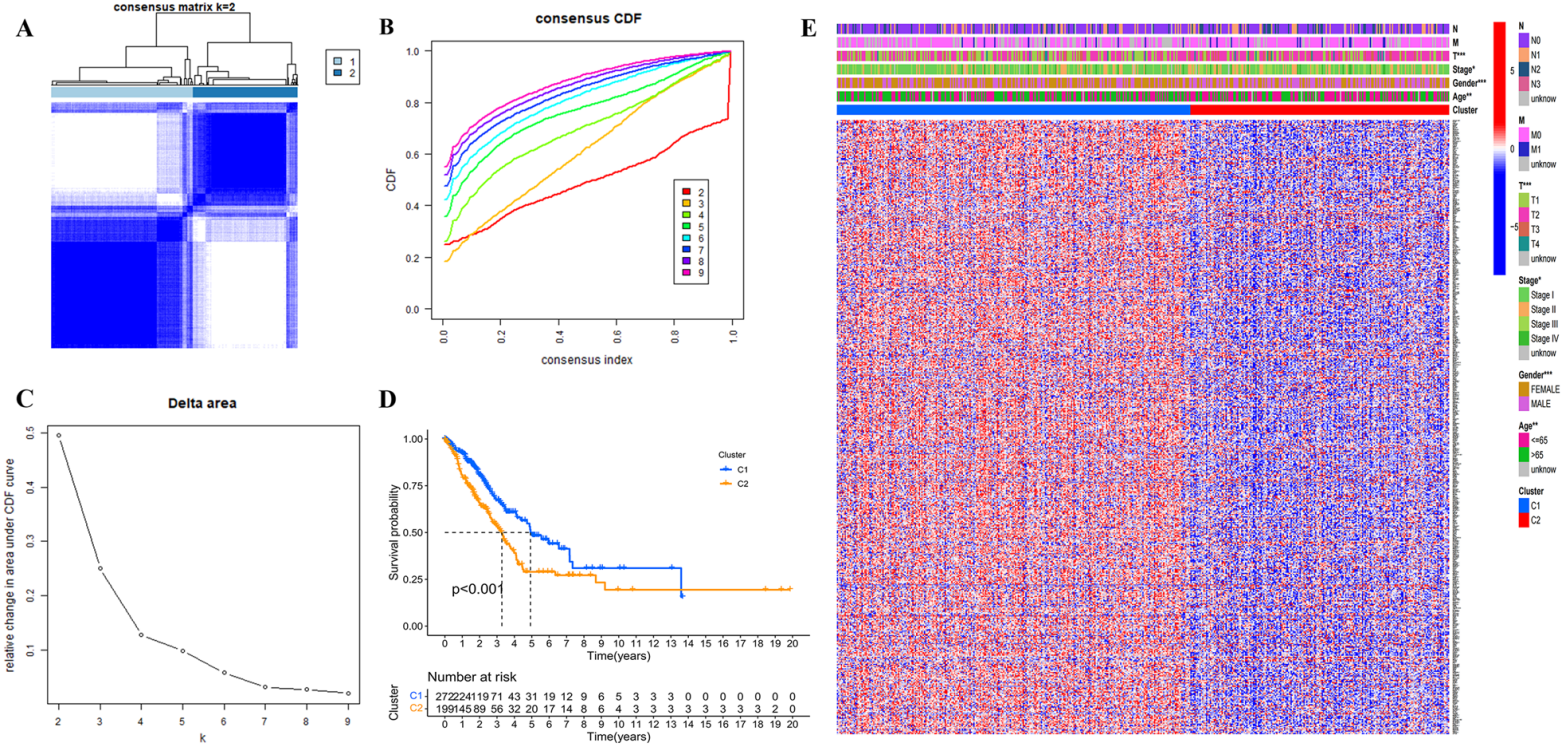
Different pyroptosis-related gene (PRGs) subtypes and clinicopathological and biological characteristics of two distinct subtypes of lung adenocarcinoma (LUAD) samples divided by clustering. Grouping of LUAD patients according to different expression of PRGs. When *k* = 2, the samples were more likely to be clustered together (**A**−**C**); multivariate analysis demonstrated differences in survival probability between two subtypes (**D**). A heatmap of clinical characteristics for patients in two subtypes (**E**)

The K-means clustering algorithm was used to classify all patients according to the differential expression of PRGs (Additional file 1). The patients with LUAD were divided into two groups (Fig. 1A–C). Information on the clinical characteristics of patients in two groups is presented by a heatmap (Fig. 1E). Results of survival analysis revealed that cluster 1 (C1) had a higher overall survival rate than cluster 2 (C2) (Fig. 1D).

### Analysis of the novel prognostic signature and establishment of the prediction model

**Fig. 2:**
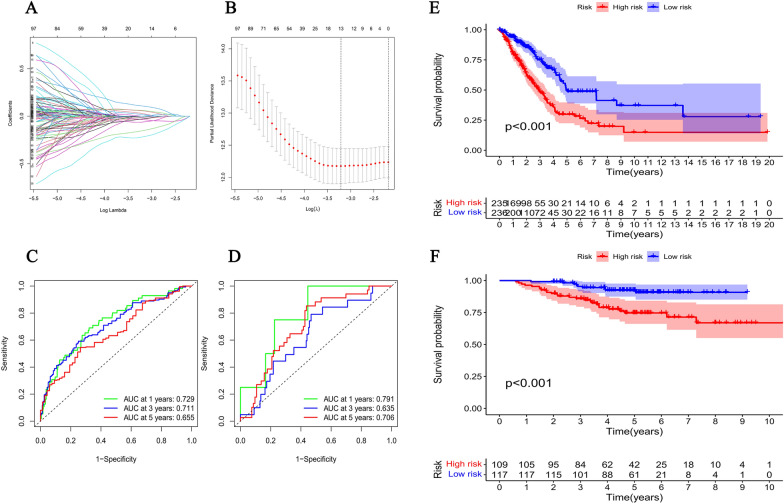
Predicting the prognosis of patients with two subtypes based on differential gene expression. Lasso regression model of lung adenocarcinoma (LUAD) patients in the TCGA cohort (**A** − **B**); the ROC curve showed the predictive efficacy of risk score to predict patient prognosis in the TCGA cohort (**C**); the ROC curve showed the predictive efficacy of risk score to predict patient prognosis in the GEO cohort (**D**); multivariate analysis demonstrated differences in survival probability between two subtypes in the TCGA cohort (**E**); multivariate analysis demonstrated differences in survival probability between two subtypes in the GEO cohort (**F**)

After grouping based on the differences in expression levels of 52 PRGs, we identified a total of 13 PRGs-related genes associated with prognosis between the two groups. Then, a model of risk score was built based on 13 PRGs-related genes, including IL-1A, P2RX1, GSTM2, ESYT3, ZNF682, KCNF1, STK32A, HHIPL2, GDF10, NDC80, GSTA1, BCL2L10, and CCR2, to explore their prognostic value. The genes we identified were differentially expressed and had significant characteristic value for the classification of LUAD subtypes, which we examined using the LASSO algorithm (Fig. 2A, B). A mathematical model on risk score was obtained using the multivariate COX regression method. The equation is as follows: Risk Score = 0.1077*IL-1A + (− 0.0146* P2RX1) + (− 0.0630* GSTM2) + (− 0.0344* ESYT3) + (− 0.1546* ZNF682) + 0.0292* KCNF1 + (− 0.0316* STK32A) + 0.0186* HHIPL2 + (− 0.0115* GDF10) + 0.0609* NDC80 + (− 0.0298* GSTA1) + 0.0571* BCL2L10 + (− 0.2199* CCR2). We calculated the risk score for all patients in the TCGA cohort. All patients were divided into high-risk and low-risk groups using the median as the cut-off value. The result of prognostic analysis showed that patients in the low-risk group have a longer life expectancy (Fig. 2E). The area under the curve (AUC) of the receiver operating characteristic (ROC) curve confirmed the reliability of predicting patient prognosis based on the risk score (Fig. 2C, AUC at 1 year = 0.729, AUC at 3 years = 0.711, AUC at 5 years = 0.655). Then, we verified the predictive value of the model with the GEO cohort. The association between risk score and life expectancy is consistent with the previous result (Fig. 2D, F, AUC at 1 year = 0.791, AUC at 3 years = 0.635, AUC at 5 years = 0.706).

**Fig. 3:**
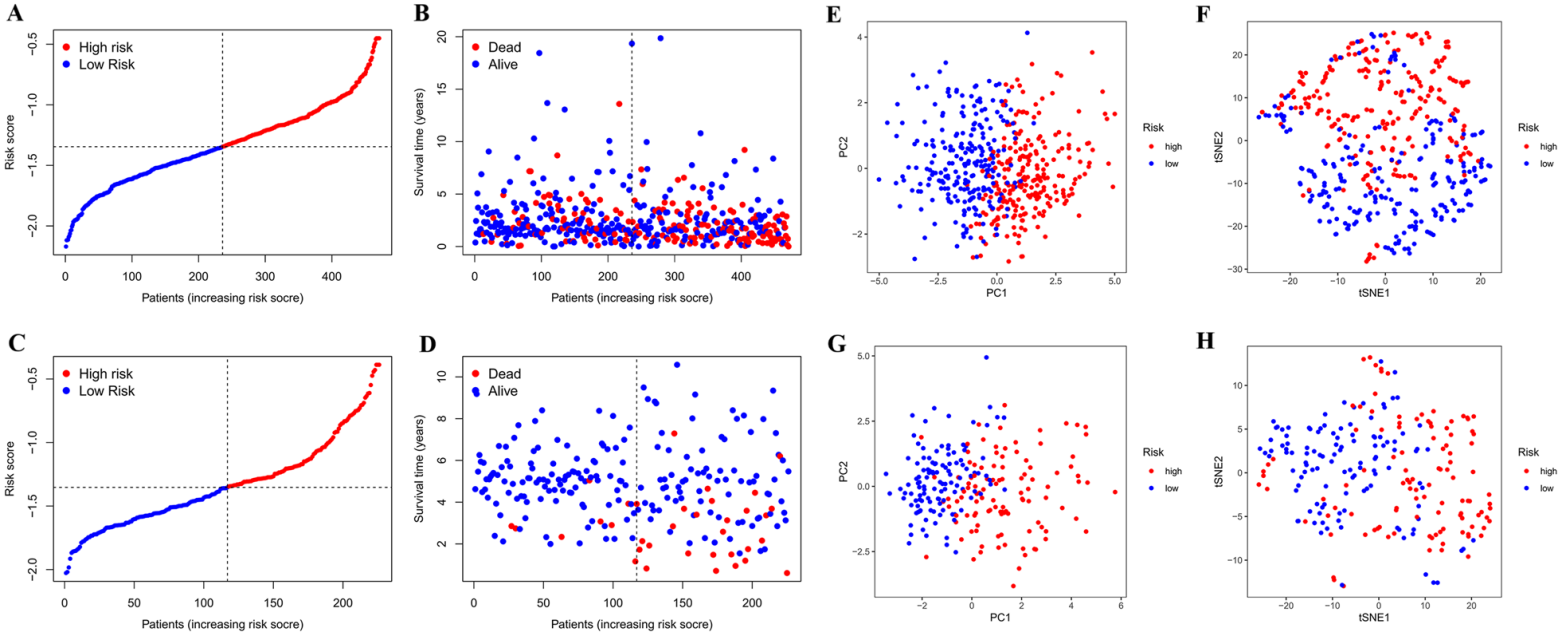
Prognostic analysis of the prediction model based on 13 genetic signature in the TCGA (**A** − **B**, **E** − **F**) and the GEO (**C** − **D**, **G** − **H**) cohorts. Risk score of lung adenocarcinoma (LUAD) patients with different subtypes in the TCGA and the GEO cohorts (**A**,**C**); distribution of survival for lung adenocarcinoma (LUAD) patients with different subtypes in the TCGA and the GEO cohorts (**B**,**D**); plots of principal component analysis (PCA) for lung adenocarcinoma (LUAD) patients with different subtypes in the TCGA and the GEO cohorts (**E**,**G**); examination of the t-SNE coefficients for lung adenocarcinoma (LUAD) patients with different subtypes in the TCGA and the GEO cohorts (**F**,**H**)

We assessed the predictive value of genetic signatures in the TCGA cohort (Fig. 3A–B, E–F) and the GEO cohort (Fig. 3C–D, G–H). The median was used as the cut-off value and all patients in both cohorts were divided into two groups (high-risk and low-risk group) (Fig. 3A, C). The patients at high risk of dying earlier than those at low risk (Fig. 3B, D). The results of PCA and t-SNE analyses revealed that the patients with different risk levels were distributed in two different directions (Fig. 3E–H).

**Fig. 4:**
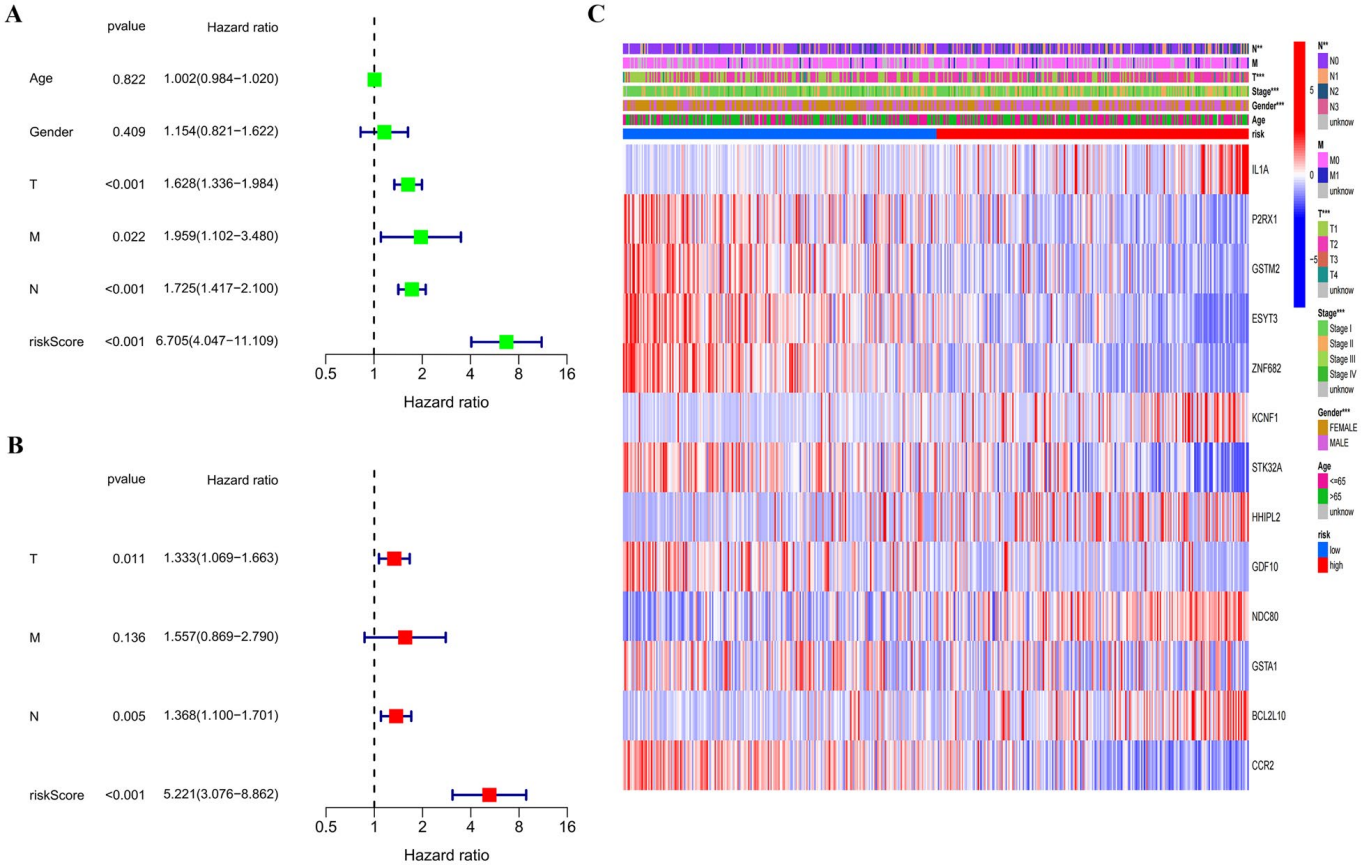
Risk factors analysis of the prediction model for lung adenocarcinoma (LUAD) patients. Univariate COX regression analysis was used to identify the variates related to the overall survival (OS) in LUAD patients (**A**); multivariate COX regression analysis was used to identify the variates related to the overall survival (OS) in LUAD patients (**B**); a heatmap of clinical characteristics for LUAD patients between the high-risk and the low-risk groups (**C**)

The univariate and multivariate COX regression analyses were used to screen independent predictors of OS in the TCGA cohort. The result of univariate COX regression analysis showed that T-stage, N-stage, M-stage, and risk score were strongly related to OS (Fig. 4A). The result of multivariate COX regression analysis showed that risk score was a significant predictor of patients’ prognosis (hazard ratio = 5.221, 95%CI 3.076–8.862, *P* < 0.001) (Fig. 4B). Next, we constructed a heatmap and found significant differences in the distribution of gender, T-stage, N-stage, and overall stage across high-risk and low-risk categories (Fig. 4C).

### Construction and validation of a nomogram with clinical features

**Fig. 5:**
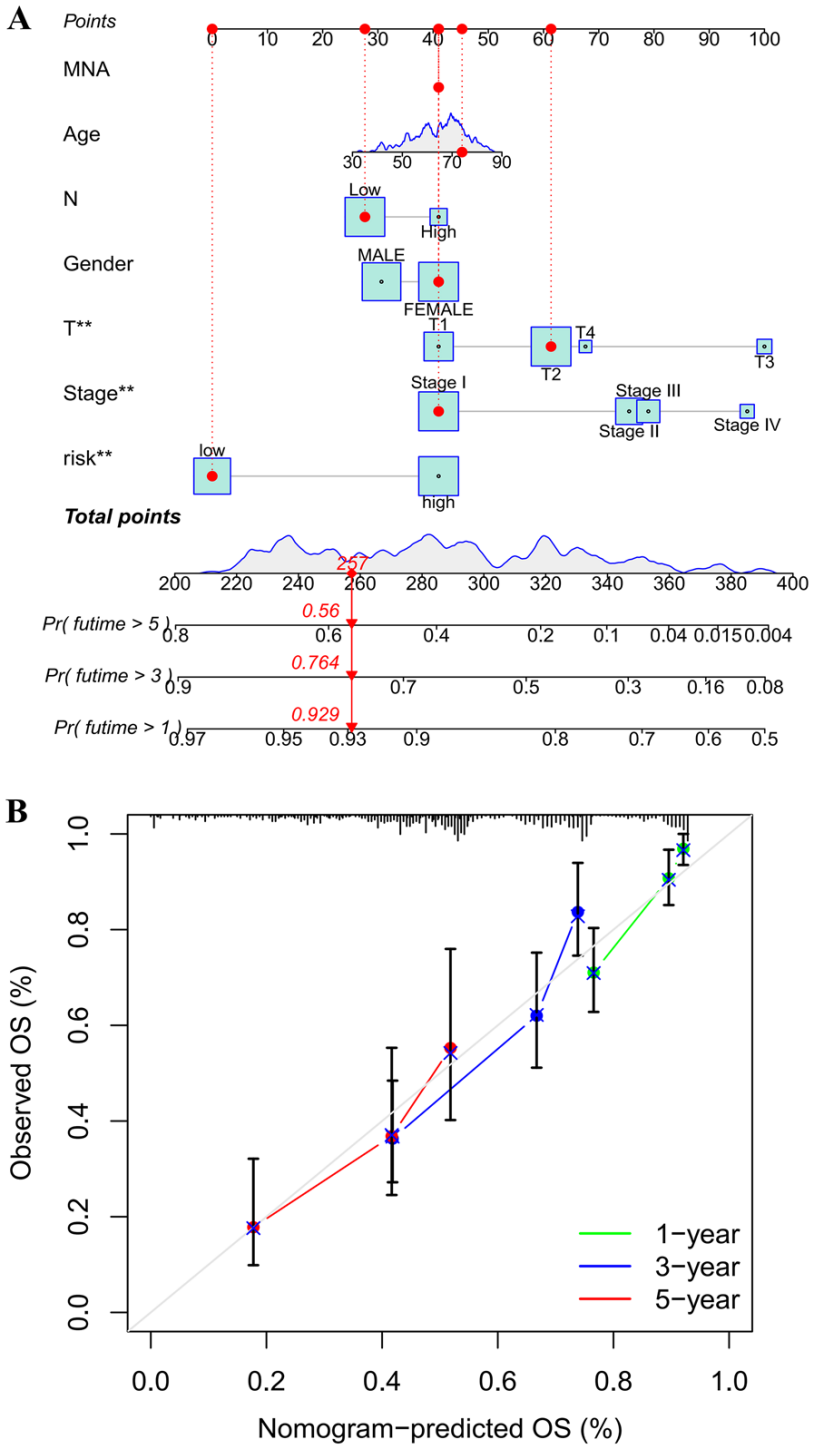
A nomogram to predict the overall survival (OS) of lung adenocarcinoma (LUAD) patients. A nomogram to predict the survival of LUAD patients in the TCGA cohort (**A**); a calibration curve for the prediction of patients’ 1, 3, 5 years OS in the TCGA cohort (**B**)

In order to visualize the prognosis of LUAD patients, we collected the information on clinical variables for all patients in the TCGA cohort and constructed a nomogram (Fig. 5A). The predictors included T stage, N stage, total stage, age, gender, and risk score. Each of the prognostic indicators in the nomogram has a corresponding point, which patients can evaluate the 1-, 3-, and 5 year survival rates according to their actual situation. Patients with higher overall points had worse clinical outcomes. The result of the calibration curve showed that the nomogram had an impressive and reliable predictive performance (Fig. 5B).

### Functional enrichment analysis based on the prediction model

**Fig. 6:**
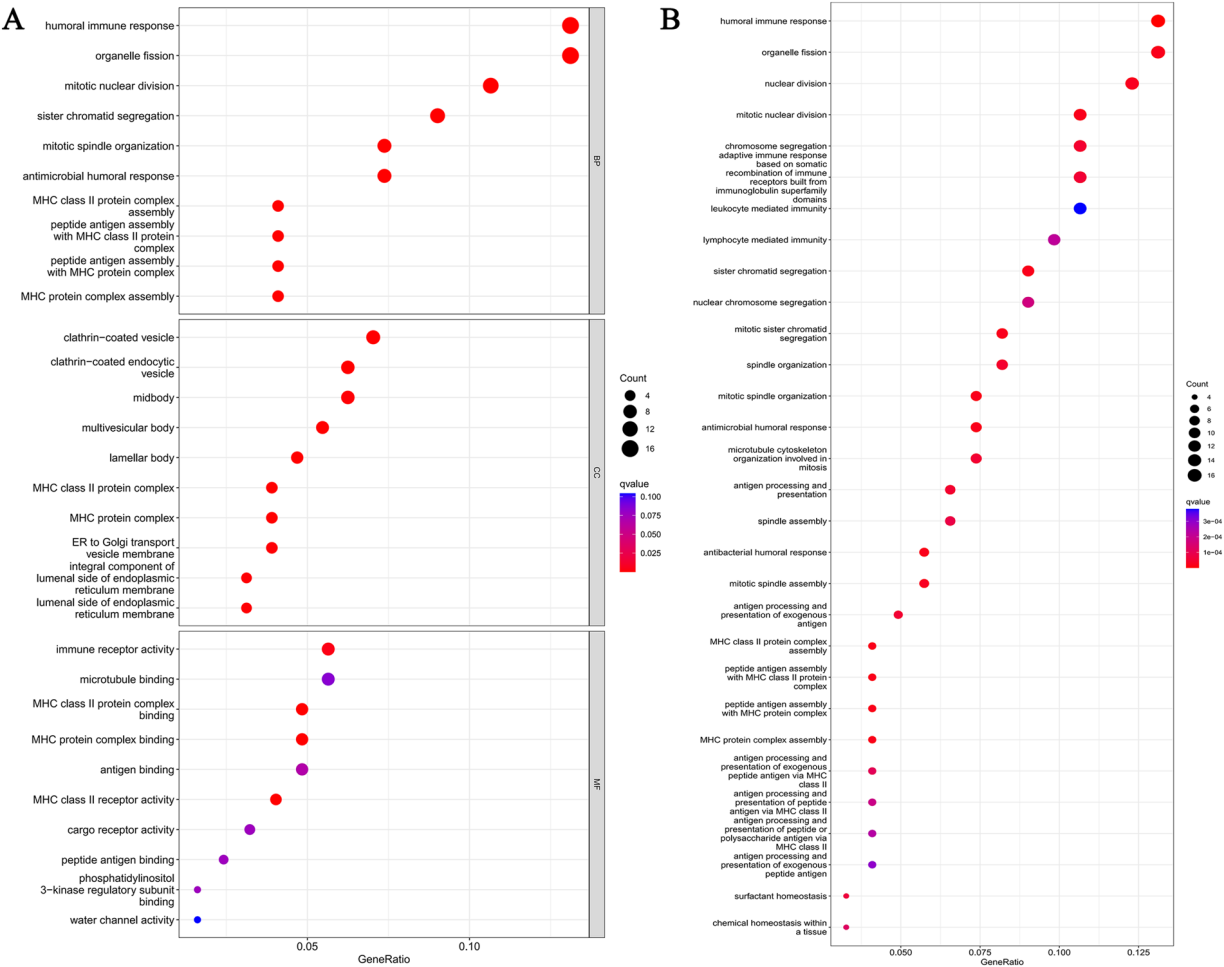
The results of GO and KEGG analyses of differentially expressed genes (DEGs) in the TCGA cohort (**A**−**B**)

GO and KEGG analyses were conducted to investigate the biological functions and pathways of DEGs. |Log_2_Fold Change|> 1 and *P*-value < 0.05 are seen as criteria. Finally, in the TCGA cohort, 122 DEGs were found in two groups divided according to differences in expression levels of 13 PRGs-related genes. Based on the DEGs, results of GO and KEGG analyses were carried out. The results showed that DEGs were mainly involved in a variety of signaling pathways related to immunology and cell differentiation, such as humoral immune response pathway, organelle fission pathway, nuclear division pathway, and mitotic nuclear division pathway (Fig. 6).

### Results of immune cells infiltration landscape analysis

**Fig. 7:**
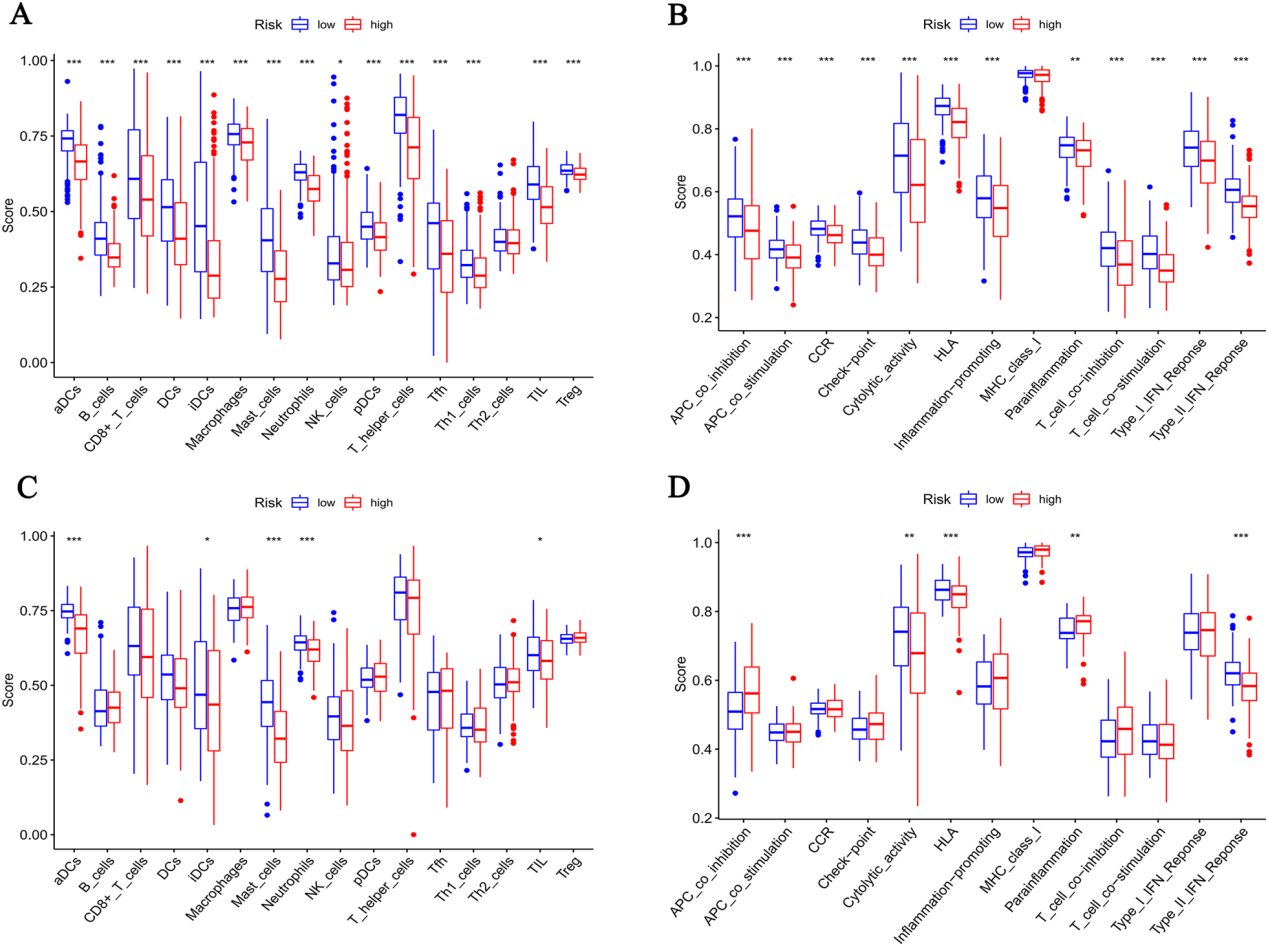
The immune status of patients in the TCGA (**A**−**B**) and GEO (**C**−**D**) cohort, including 16 different immune cells and 13 different immune-related pathways

Patients in the TCGA and GEO cohorts were analyzed separately for immune cells infiltration landscape analysis based on differences in risk score, including 16 different immune cells and 13 different immune-related pathways. The results showed that in the TCGA cohort, the amounts of ‘‘DCs’’, ‘‘B Cells’’, ‘‘CD8^+^ T Cells’’, ‘‘Macrophages’’, ‘‘Mast Cells’’ ‘‘Neutrophils’’, ‘‘NK Cells’’, ‘‘T helper Cells’’, ‘‘Tfh’’, ‘‘Th1 Cells’’, ‘‘TIL’’, and ‘‘Treg’’ were significantly higher in the low-risk group than in the high-risk group (Fig. 7A). Similarly, in the GEO cohort, the amounts of ‘‘DCs’’, ‘‘Mast Cells’’, ‘‘Neutrophils’’, and ‘‘TIL’’ were significantly higher in the low-risk group than in the high-risk group (Fig. 7C). Multiple immune-related pathways also showed more activity in the low-risk group than in the high-risk group, including APC co-inhibition, cytolytic activity, HLA, parainflammation, and type II IFN response (Fig. 7B, D). There is evidence that prognostic differences between patients in the high-risk and low-risk groups are due to the differences in immune characteristics.

## Discussion

Pyroptosis is a unique form of cell death executed by the GSDM protein family, involving multiple immune and inflammatory responses [8]. The classical pyroptosis is associated with NLRP3 inflammasome complex, which comprises NLRP3, ACS and caspase-1 [18]. Pyroptosis plays a dual role in cancer progression and therapeutic mechanisms [19]. Pyroptosis-derived cytokines can induce the transformation of normal cells into tumor cells. However, pyroptosis can also promote tumor cell death. Pyroptosis signature has been used to predict prognosis in a variety of malignancies, but the role in lung adenocarcinoma is unclear, and the aim of our study was to elucidate this role.

The current tumor-node-metastasis (TNM) staging system is important in assessing the prognosis of patients with malignancies [20]. Our results also revealed differences in the prognosis of patients with different T stages, N stages, and total stages (Fig. 5). However, it is difficult to make accurate survival predictions and treatment decisions for LUAD patients based on TNM stages. Therefore, we developed a risk scoring system (Risk Score) associated with DEGs of different PRGs subtypes in LUAD patients. In this study, assessing the expression levels of 13 PRGs-related genes in combination with traditional TNM classification can better guide survival predictions and treatment decisions for patients with LUAD (Fig. 5).

In this study, we established a prognosis prediction model for LUAD patients based on the pyroptosis-related genes (PRGs), and confirmed the validity and applicability of the model. 13 PRGs-related genes (IL-1A, P2RX1, GSTM2, ESYT3, ZNF682, KCNF1, STK32A, HHIPL2, GDF10, NDC80, GSTA1, BCL2L10 and CCR2) were found for the prognostic signature. For further understanding of the model, we searched the information on key genes.

Interleukin-1 (IL-1) is a symbol of systemic inflammation and cancer in humans. IL-1A is a member of the IL-1 family, which is widely involved in the genesis, progression, and metastasis of tumors. The expression level of IL-1A was found to be significantly increased in a variety of cancers, including non-small cell lung cancer, colon cancer, and squamous cell carcinoma [21]. Similarly, IL-1A can also promote macrophage aggregation to stimulate angiogenesis, leading to the progression and metastasis of tumor [22, 23]. Glutathione S-transferase Mu 2 (GSTM2) and Glutathione S-transferase A1 (GSTA1) are enzymes belonging to the GST family that are significant in carcinogen detoxification. GSTM2 plays an important role in the development and metastasis of lung cancer. The results of previous studies showed that GSTM2 mRNA levels were significantly lower in the tumor tissues of NSCLC patients compared to the paired adjacent normal tissues [24]. The high expression of GSTM2 is also correlated with the favorable survival of patients with lung cancer [25]. Our finding reveals that the expression level of GSTM2 is higher in patients of cluster 1 (C1), which have better clinical outcomes. GSTA1 is closely associated with metastasis in lung cancer. Overexpression of GSTA1 can mediate lung cancer cells metastasis by promoting epithelial–mesenchymal transition (EMT) [26]. The expression level of GSTA1 is also an important predictive factor associated with postoperative recurrence in NSCLC patients [27]. The expression level of GSTA1 is higher in patients of cluster 2 (C2), which have worse clinical outcomes. Serine/threonine kinase 32A (STK32A) has been confirmed by epidemiological investigations as a susceptibility gene for lung cancer [28]. Overexpression of STK32A enhances migration and proliferation of lung cancer cells while inhibiting apoptosis, which is essential for lung cancer progression [29]. However, miR-130a-5p can inhibit the expression of STK32A by regulating RUNX2 to suppress the above process. C–C motif chemokine receptor 2 (CCR2) encoded protein is a chemokine that specifically mediates monocyte chemotaxis. It is involved in monocyte infiltration in inflammatory diseases and as well as in the inflammatory response against tumors. The inflammatory microenvironment is a key factor contributing to lung cancer progression. Tumor-associated macrophages (TAMs) are important components of the inflammatory microenvironment [30]. Evidence reveals that M2-polarized TAMs play an important role in the progression and metastasis of lung cancer [31]. It has been found that estrogen receptor α (Erα) can activate the CCL2/CCR2 axis to promote macrophage infiltration, M2 polarization, and MMP9 production, which can then increase NSCLC cell invasion [32]. Significant correlations were found among the higher expression of CCR2 and the worse pathological stage and the shorter OS of LUAD patients [33]. Therefore, interventions of CCR2 expression and M2 polarization TAMs may be potential options for the treatment of lung cancer.

Our research categorizes the LUAD patients based on differential expression levels of PRGs, discovers the DEGs between different subtypes, and establishes a nomogram to identify the relationship between pyroptosis and patients’ prognosis. The significance of pyroptosis-mediated immunophenotype in the occurrence, development, and prognosis of LUAD was also systematically revealed. The prediction model we have developed can be a powerful tool for predicting the prognosis of different subtypes of LUAD. However, this study remains some limitations. First, we only used the datasets from the TCGA and GEO databases for the analysis, more data from different regions are needed for validation. Furthermore, due to the limited information contained in the databases, the predictive model cannot be well used to guide the clinical treatment of patients with different subtypes of LUAD. Finally, further in vivo and in vitro experiments are needed to validate the results.

### Supplementary Information

**Additional file 1.** The process of K-means clustering algorithm.

## Data Availability

All data can be found in TCGA and GEO databases. Ethical approval has been obtained for this study.
