# Supplementary figures and images for: Risk factors analysis and survival prediction model establishment of patients with lung adenocarcinoma based on different pyroptosis-related gene subtypes
Source: Eur J Med Res. 2023 Dec 18;28:601. doi: 10.1186/s40001-023-01581-x (PMC10726488; doi:10.1186/s40001-023-01581-x)

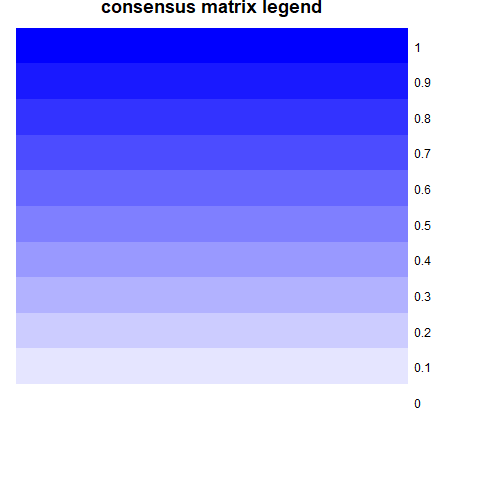

Supplement: Supplementary file 1 — Additional file 1. The process of K-means clustering algorithm. [file 40001_2023_1581_MOESM1_ESM.zip › Additional file 1/consensus001.png]

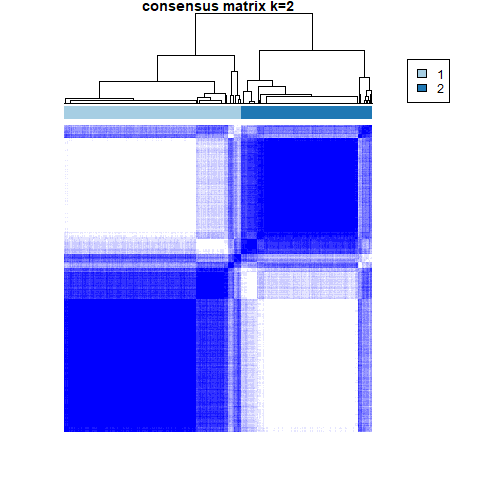

Supplement: Supplementary file 1 — Additional file 1. The process of K-means clustering algorithm. [file 40001_2023_1581_MOESM1_ESM.zip › Additional file 1/consensus002.png]

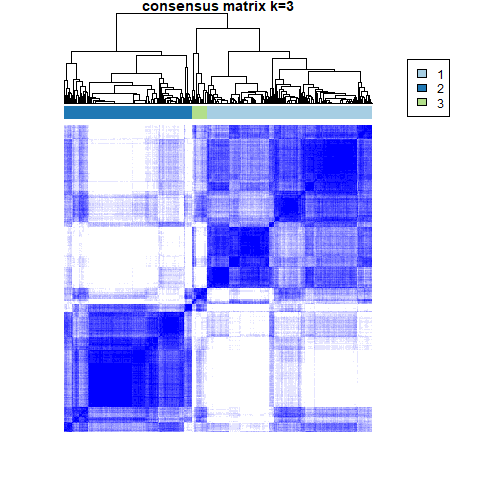

Supplement: Supplementary file 1 — Additional file 1. The process of K-means clustering algorithm. [file 40001_2023_1581_MOESM1_ESM.zip › Additional file 1/consensus003.png]

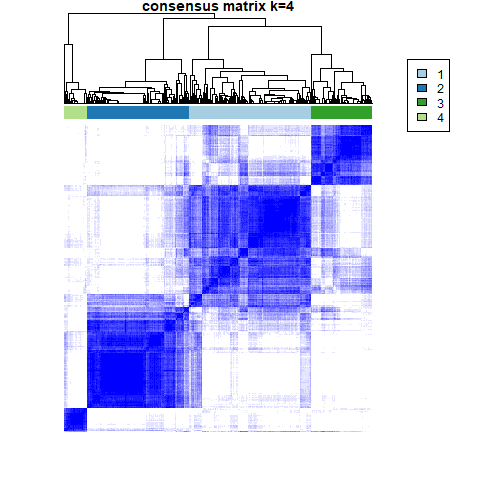

Supplement: Supplementary file 1 — Additional file 1. The process of K-means clustering algorithm. [file 40001_2023_1581_MOESM1_ESM.zip › Additional file 1/consensus004.png]

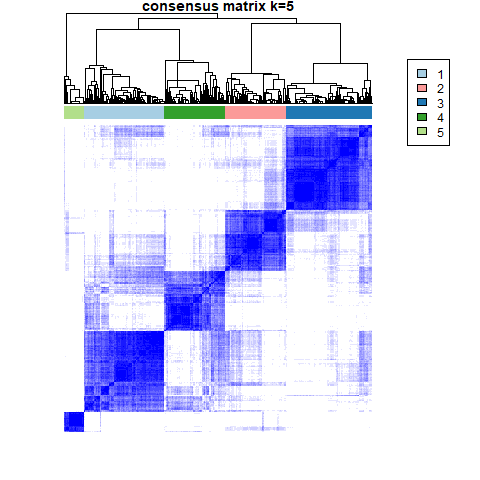

Supplement: Supplementary file 1 — Additional file 1. The process of K-means clustering algorithm. [file 40001_2023_1581_MOESM1_ESM.zip › Additional file 1/consensus005.png]
